# The Effects of Graphene Oxide-Silica Nano-Hybrid Materials on the Rheological Properties, Mechanical Properties, and Microstructure of Cement-Based Materials

**DOI:** 10.3390/ma15124207

**Published:** 2022-06-14

**Authors:** Zizhi Long, Youzhi Chen, Weisong Yin, Xiuqi Wu, Yun Wang

**Affiliations:** 1 State Key Laboratory of Silicate Materials for Architectures, Wuhan University of Technology, Wuhan 430070, China; 290873@whut.edu.cn (Z.L.); cyzly@whut.edu.cn (Y.C.); 303879@whut.edu.cn (X.W.); 2 Key Laboratory of Roadway Bridge & Structure Engineering, Wuhan University of Technology, Wuhan 430070, China; 3 Hubei Hongyu Qiaolene Material Technology Co., Ltd., Wuhan 430070, China; wyun@whut.edu.cn

**Keywords:** graphene oxide, nanomaterials, cement-based materials, dispersity, fuzzy matric analysis

## Abstract

Despite their excellent performance, two-dimension nanomaterials have certain limitations in improving the performance of cement-based materials due to their poor dispersity in the alkaline environment. This paper has synthesized a new two-dimension stacked GO-SiO_2_ (GOS) hybrid through the sol-gel method. Nano-SiO_2_ is coated on the surface of GO with wrinkling characteristics, and the atomic ratio of C, O, and Si in GOS is 1:1.69:0.57. The paper discusses the impacts on the spreading, Marsh cone flow time, rheological properties, mechanical properties, and microstructure of cement-based materials for the GOS at different mixing quantities. Furthermore, with the same mixing quantity of 0.01%, the influences on the dispersity, flow properties, rheological parameters, and mechanical properties of GOS and graphene oxide (GO) are compared. Lastly, fuzzy matrix analysis has been adopted to analyze the comprehensive performance of cement-based materials containing GOS. The research results indicate that, compared with the reference sample, the spreading for the GOS cement mortar with 0.01% mixing quantity was reduced by 4.76%, the yield shear stress increased by 37.43%, and the equivalent plastic viscosity was elevated by 2.62%. In terms of the 28 d cement pastes, the compressive and flexural strength were boosted by 27.17% and 42.86%, respectively. According to the optical observation, GOS shows better dispersion stability in the saturated calcium hydroxide solution and simulated pore solution than GO. Compared with the cement-based materials with the same mixing quantity (0.01%), GOS has higher spreading, lower shear yield stress, and higher compressive and flexural strength than GO. Finally, according to the results of fuzzy matrix analysis, when the concentration of GOS is 0.01%, it presents a more excellent comprehensive performance with the highest score. Among the performance indicators, the most significant improvement was in the flexural properties of cement-based materials, which increased from 8.6 MPa to 12.3 MPa on the 28 d.

## 1. Introduction

For the past few years, two-dimension nano graphene oxide (GO) has optimized the microstructure of cement-based materials from the nanoscale. Improving such materials’ comprehensive performance has become a research focus [1]. Many studies [2,3] show that GO can promote/regulate/catalyze the hydration of cement for its size effect, high specific surface area, and rich oxygen-containing functional groups on the surface. Its high specific surface area provides more valid nucleation sites for the crystallization nucleation of hydration products, thus effectively regulating the morphology and size distribution of the crystals of hydration products after the hardening of the cement-based materials. Meanwhile, the rich oxygen-containing functional groups on the surface can be firmly combined with the hydration products. This “bridging effect” allows the hydration products to make contact with the formed network earlier, improving the macro-mechanical properties of the cement-based materials [4,5,6]. Indukuri et al. [7] added 0.03% GO to increase such materials’ flexural and compressive strength by 77.70% and 47.61%, respectively. According to the scanning electron microscope (SEM), GO effectively decreases the voids and defects in cement-based materials, making the arrangement of hydration products tighter.

Similar to other nanomaterials, the addition of GO can be averse to the flowability and rheology of cement-based materials. On the one hand, the magnificent specific surface area of GO absorbs considerable free water and compromises its performance. On the other hand, with the Van der Waals forces between layers, the active functional groups of GO are susceptible to interacting with the ions in pore fluid, lowering the performance of cement pastes because of the reduction of the distance between cement particles and the increase of the frictional resistance among cement particles. Through methods like SEM, Fourier transform infrared spectroscopy (FT-IR), and X-ray fluorescence spectrum (XRF), Wang et al. [8] proved that GO has powerful absorption capacity on the surface of cement particles. With the Van der Waals forces, the carboxyl on the surface reacts with the metal cation and makes the cement particles come closer, thus reducing the fluidity of such pastes. Lu et al. [9] believed that GO agglomerates in the stirring process, wrapping a large amount of free water, which can significantly reduce the fluidity even at a very small amount. Pan et al. [10] discovered that the spreading of cement pastes with 0.05 wt% of GO decreased by 41.7% compared with the reference sample.

To improve the adverse influences of GO on the performance of cement-based materials, Shang et al. [11] cladded the GO on the surface of silica fume for modification to form GOSF composites; these composites were mixed into cement-based materials. Compared with the SF equivalent alternative cement sample, if the mass for the GOSF alternative cement was 8%, the yield stress and plastic viscosity decreased by 39.6% and 18.2%, respectively. By adding fly ash (FA), Wang et al. [12] increased the fluidity of cement pastes with GO. The results indicate that the yield stress and plastic viscosity reduced with the increase of FA. Li et al. [13] conducted mechanical separation of GO through silica fume and analyzed the microstructure and mechanical properties. According to the results, the addition of silica fume significantly improved the dispersity of GO nanosheets. As a result, the wrapping of free water was reduced. Although these methods have effectively improved the performance of cement-based materials to different extents, they still have certain limitations as they fail to realize the modification of GO molecules.

GO-SiO_2_ nanohybrids have been used in many fields. Liu et al. have shown that GO-SiO_2_ can be well dispersed in polyacrylate (PA) [14]. In addition, the improvement of mechanical properties of carriers by GO-SiO_2_ has also been widely reported [15,16]. Therefore, this paper synthesized a new two-dimension nanohybrid GO-SiO_2_ (GOS) with GO and nano SiO_2_ through the sol-gel method. Transmission electron microscopy (TEM), X-ray diffraction (XRD), X-ray photoelectron spectroscopy (XPS), Raman spectroscopy, and Fourier-transform infrared (FT-IR) spectroscopy were adopted for characterization. The dispersity and stability in the simulated cement pore fluid for GOS and GO were compared and analyzed. The paper discusses the impacts on spreading, Marsh cone flow time, rheological properties, and mechanical properties while further exploring the microstructures of cement-based materials through XRD, thermogravimetry, and SEM for the GOS at different mixing quantities. Furthermore, the influences on the rheological parameters and mechanical properties for GOS and GO were compared and studied with the same mixing quantity. Lastly, fuzzy matrix analysis was adopted to evaluate and analyze the comprehensive performance of the cement-based materials containing different mixing quantities of GOS.

## 2. Materials and Test Methods

### 2.1. Materials

The chemical substances applied in the research are graphene (≤44 μm), concentrated sulfuric acid (H_2_SO_4_, 98%), hydrochloric acid (HCl, 38%), potassium permanganate (KMnO_4_), sodium nitrate (NaNO_3_), hydrogen peroxide (H_2_O_2_, 30%), ammonia solution (28 wt%) and tetraethyl orthosilicate (TEOS, 99%). These reagents are purchased from Shanghai National Pharmaceutical Group Chemical Reagent Co., Ltd. (Shanghai, China). Ordinary Portland cement (P.O 42.5) made by Wuhan Huaxin Cement Co., Ltd. (Wuhan, China) was adopted to prepare the cement-based materials. The content of cement composition measured by X-ray fluorescence (XRF) is shown in Table 1. The solid content of the polycarboxylate superplasticizer (PC) is 35%, and it was diluted to 20% while applying.

### 2.2. The Preparation and Characterization of GO and GOS

The improved Hummers method was adopted to prepare the GO [17], and the specific preparation process consists of three steps: oxidation, purification, and stripping. Concentrated sulfuric acid and potassium permanganate were utilized as oxidizing agents to transfer the expandable graphite powder into the GO in low-temperature, medium-temperature, and high-temperature environments. Then, the deionized water was added to the oxidized products to remove the impurities through centrifugation and dialysis. Then, the GO was stripped through ultrasonic oscillation, and the aqueous solution of GO could be obtained by expanding its interlayer spacing. According to the experimental process in the literature [18], the silica particles can be deposited on the GO sheet through the in situ hydrolysis and dehydration condensation of TEOS, obtaining the nano hybrid of GO-SiO_2_ (GOS). The specific steps are as follows: Through 0.5 h of ultrasonic processing, disperse 300 mg of GO into 75 mL of ethanol solution. After that, regulate the pH of the solution to about 9 with a small amount of ammonia solution and conduct 1 h of the ultrasonic processing. Afterward, add 1.4 mL TEOS solution and stir for 48 h at the constant temperature of 40 °C; hold the mixture still at room temperature (25 °C) for 48 h. Then, remove the free silica in the nanohybrid suspension of GOS through centrifugation and dialysis. Finally, dry the GO and GOS aqueous solution for 12 h in the vacuum environment at 50 °C, then grind the mixture to a powder.

Next, 0.05 mg/mL of GO and the GOS ethanol solution was extracted with copper mesh and put into the transmission electron microscope (TEM: working voltage 120 KV and model are JEM-1400Plus) after being dried at room temperature to observe the surface appearance. The X-ray photoelectron spectrometer (XPS), X-ray diffractometer (XRD), ultraviolet spectrophotometer (UV-VIS), and infrared spectrometer were adopted to represent the degree of oxidation of GO and the insertion of the GO and GOS functional groups.

### 2.3. The Preparation of Cement-Based Materials

**Figure 1:**
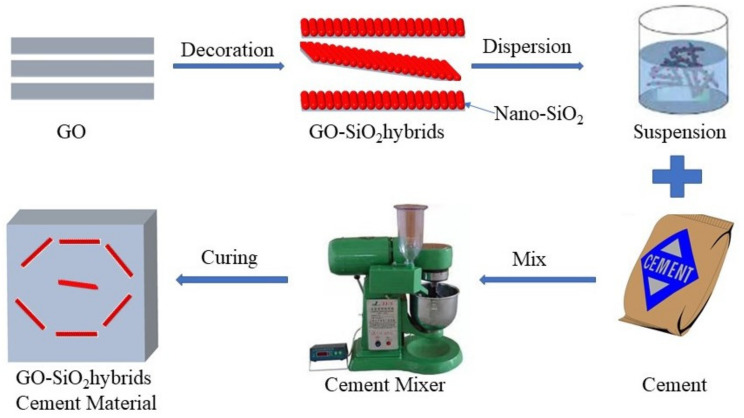
GOS cement-based material preparation process.

Three types of cement-based composites were prepared. The first is a cement slurry without nanomaterials, marked as a control, as a reference. The second is the cement paste with 0.01%GO, marked as GO1, and the third is the addition of 0.01%, 0.03%, 0.05% and 0.07% (cement mass ratio) of GOS, respectively, marked as GOS1, GOS3, GOS5 and GOS7. First, PC and water were separately added to the prepared GO and GOS to mix, and then the mixture underwent ultrasonic processing for 30 min; next, the ultrasonically dispersed liquid was added to the cement and rapidly stirred at a rate of 280 rpm for 2 min. Then, the mixture was slowly mixed for 1 min at 140 rpm [19]. Please refer to Table 2 for the detailed proportions of cement-based material. Figure 1 displays the flow chart of the production. After the fluidity and rheological parameters were tested, the cement pastes were put into molds with the size of 40 mm × 40 mm × 160 mm and 40 mm × 40 mm × 40 mm. Curing was conducted under the standard conditions (100% RH, 25 °C) to test the compressive and flexural strength after different periods (3, 7, and 28 days).

### 2.4. Test of Dispersion Stability

The stability of GO and GOS nanomaterials in the deionized aqueous solution, saturated Ca (OH)_2_ solution, and simulated OPC pore solution were studied. The ion concentration in the simulated solution was similar to the one extracted from the cement pastes. Table 3 lists the chemical components of the simulated OPC pore solution [20,21]. The dispersion stability of the suspension liquid at 0 h, 6 h, 24 h, and 3 d was observed through macro-optics.

### 2.5. Mini-Slump Flow Diameter

Following EN 1015-3 [22], for the measurement of the fluidity of cement pastes, a jointless truncated cone round die with a smooth internal wall (top diameter is 36 mm, lower diameter is 60 mm, and height is 60 mm) was adopted to measure the spreading of the cement pastes with GO or GOS.

### 2.6. Marsh Cone Flow Time

The Marsh cone test is an experiment to test the performance of cement pastes. The Marsh cone with a volume of 1200 mL and a diameter of 5 mm was adopted in this research. A total of 1000 mL of neat pastes were poured into the Marsh cone to test the time to flow out.

### 2.7. Test of Rheological Properties

A rheometer (R/S SST 2000 instrument) made by Mingkesi in China was adopted in the research to measure the rheological properties, including viscosity and shear stress, of the cement pastes. The specific test procedures are described as follows: The rheological test began from the prestressing, and the shear rate was maintained at 100^−1^ for 60 s to ensure the evenness and stability of the pastes; next, the shear rate was increased from 0 to 100 s^−1^ in 60 s and decreased from 0 s^−1^ to 100 s^−1^ in 60 s. The cycle was repeated twice, and the total test duration was 300 s. The Herschel-Bulkley (H-B) model was adapted to fit the rheological curve. The H-B Equation (1) was adapted to estimate the shear yield stress, and Equation (2) was utilized to estimate equivalent plastic viscosity:

(1) $$\tau =\tau_{0}+k\gamma^{n}$$

(2) $$\mu_{p}=\frac{3k}{n+2}\gamma_{max}^{n-1}$$

where *τ* is shear stress (Pa), *k* is the coefficient of consistency, *γ* is the shear rate (s^−1^), *n* is a rheological index (shearing thinning, *n* < 1; shearing thickening, *n* > 1), and *μ_p_* is the equivalent plastic viscosity.

### 2.8. Test of Mechanical Properties

After the flow and rheological properties were tested, all the cement pastes were poured into the die covered with plastic film and demolded after 1 d. The samples of cement-based materials were maintained at 3 d, 7 d, and 28 d under the standard conditions, and then the flexural and compressive strength tests were performed according to EN1015-11 [23]. To alleviate errors, all tests were conducted three times.

### 2.9. Microscopical Test

XRD and synthetical thermal analyzer (TG-DSC) were adopted to analyze the micro performance of the cement-based materials. Field emission scanning electron microscopy was utilized to represent the microstructures of the hydration products. The cement matrix that had been cured for 28 days was broken, and core fragments with a size of 5 mm × 5 mm × 2 mm were taken and immersed in ethanol for 24 h to terminate the hydration. Next, they were put into a vacuum drying oven with a temperature of 60 °C for 24 h, which were then ground to a size that could pass through the 200-mesh sieve. These particles were taken as the samples for the XRD and TG-DSC analysis. In addition, in terms of the TG-DSC, the contents of chemically bound water (BW) and calcium hydroxide (CH) were calculated through Equations (3) and (4):

(3) $$\mathrm{BW}\left(\%\right)=W_{105}-W_{800}$$

(4) $$\mathrm{CH}\left(\%\right)=\frac{74}{18}W_{\mathrm{CH}}+\frac{74}{44}W_{{\mathrm{CaCO}}_{3}}$$

where:

W_105_—the mass fraction of residual samples when heated to 105 °C (%);

W_800_—the mass fraction of residual samples when heated to 800 °C (%);

W_CH_—the mass fraction of CH water loss when heated to 400–500 °C (%);

$W_{{\mathrm{CaCO}}_{3}}$—the mass fraction lost by the decomposition of CaCO_3_ when heated to 600–700 °C (%).

## 3. Results and Discussion

### 3.1. GO and GOS Nanohybrids Characterization

**Figure 2:**
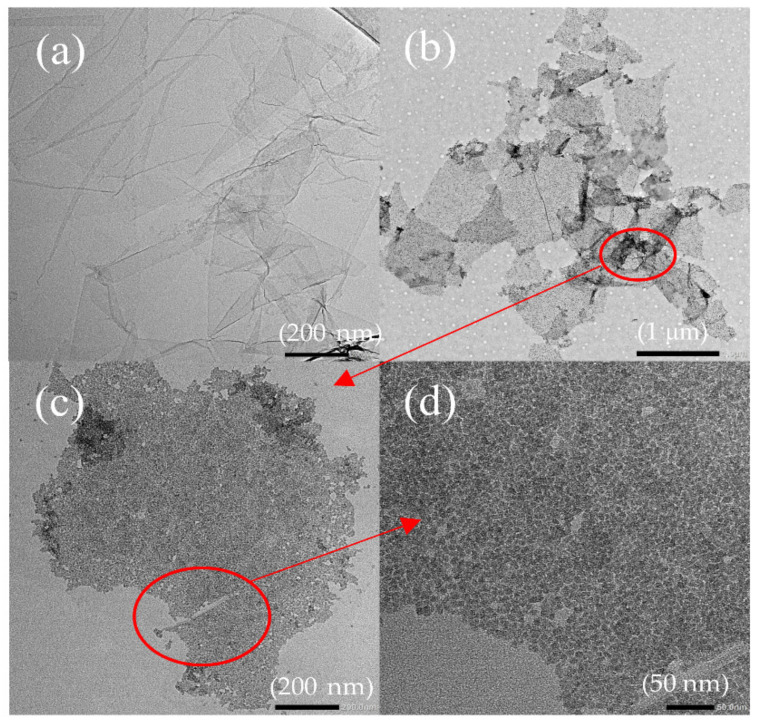
TEM images of GO and GOS. (**a**) GO. (**b**) GOS. (**c**) Locally enlarged images of GOS in (**b**). (**d**) Locally enlarged images of GOS in (**c**).

Figure 2a shows the TEM image of GO lamella in the GO aqueous solution of 0.05 mg/mL. It offers a vast plane, and the two-dimension structure of GO makes the visual effect visible. The prepared GO is semitransparent with distinct folds. Figure 2b displays the TEM image of GOS lamella in the GOS ethanol solution of 0.05 mg/mL. Figure 2b–d shows the attachment of SiO_2_ on GO, where the GO substrate containing fold features is clearly visible in Figure 2b, and Figure 2c,d is enlarged images of Figure 2b. The black dots in Figure 2c represent silicon dioxide. As can be seen, they are evenly overlapped on the GO lamella. In addition, the sol-gel reaction between the TEOS and the GO shows no impact on the two-dimension structure of GO, which is of great significance to the application of GO in cement-based materials.

**Figure 3:**
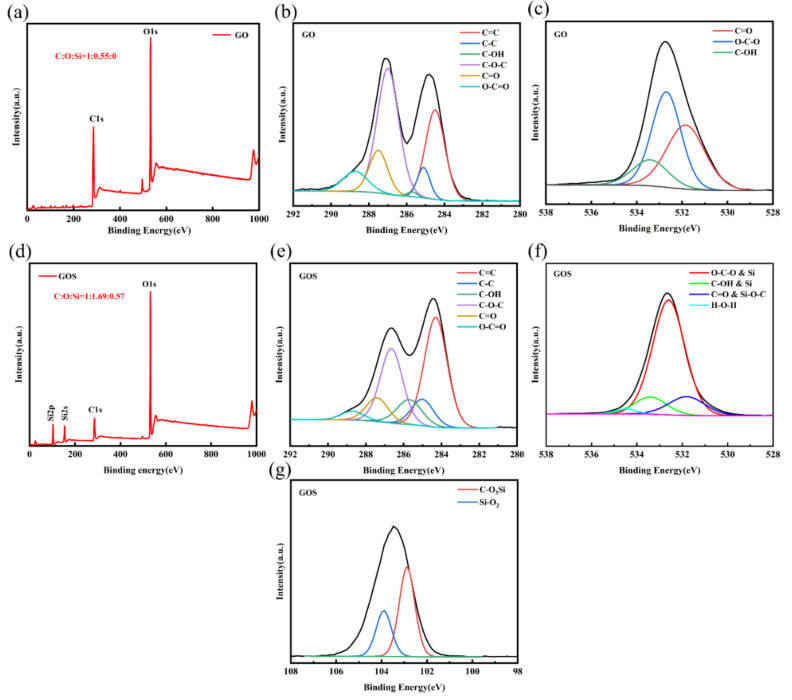
The XPS characterization spectra: (**a**) The GO full spectrum. (**b**) The GO C1s deconvolution peak. (**c**) The GO O1s deconvolution peak. (**d**) The GOS full spectrum. (**e**) The GOS C1s deconvolution peak. (**f**) The GOS O1s deconvolution peak. (**g**) The GOS Si2p deconvolution peak.

XPS characterization can be used to determine the adhesion of SiO_2_ to GO. According to the XPS spectral characteristics in Figure 3, the C/O ratio of GO is 1.81, which indicates that the oxidation degree of GO is very high. The deconvolution of the C1s and O1s spectra of GO shows that the peaks of the oxygen-containing functional groups 284.1, 285.4, 285.9, 286.5, 287.2, and 288.6 eV correspond to C=C, C-C, hydroxyl (C-OH), epoxy ether (C-O-C), carbonyl(C=O), and carboxylate (O-C=O), respectively. According to Figure 3d–g, the C/O ratio of GOS is 0.59. This is because the attachment of SiO_2_ increases the content of oxygen atoms. The oxygen atom of GO in GOS is subtracted, and its O/Si ratio is 2, which confirms that the attachment on GO is SiO_2_. According to Figure 3f,g, the peaks at about 531.8, 533.4, and 103.9 eV indicate the formation of SiO_2_, while the peaks at 531.8 and 102.8 eV are evidence of covalent bond formation between GO and SiO_2_. In addition, compared with the C1s peaks of GO and GOS, the intensity of the GOS peak related to the oxygen-containing functional groups on the right side decreases, which proves that the oxygen-containing functional groups on the surfaces of SiO_2_ and GO are bonded. Thus the peak strengths of C-OH, C-O-C, C=O, and O-C=O in Figure 3e are reduced [24].

**Figure 4:**
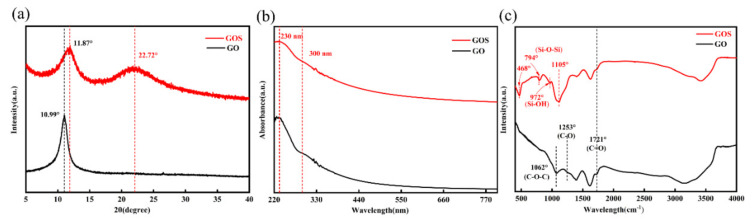
(**a**) The XRD spectra of GO and GOS. (**b**) The UV–vis spectra of GO and GOS in water. (**c**) The FTIR spectra of GO and GOS.

The XRD diffraction diagram of GO and GOS is shown in Figure 4a. GO has a significant diffraction peak when 2θ is 10.99°, corresponding to the (001) graphite oxide phase, consistent with the literature [25]. The synthesized interlayer spacing of GO is 0.804 nm, indicating that many oxygen-containing functional groups have been inserted into the interlayer of graphite with severe oxidization. In the XRD diffraction diagram of GOS, the angle of the characteristic diffraction peak for GO has shifted from 10.99° to 11.89°. This shift is because the dehydrating condensation of the hydroxyl and carboxyl groups on the TEOS with GO prevented GOS from re-stacking. Furthermore, due to a load of amorphous silica particles, a wide peak appeared when 2θ = 22.72° for the GOS image in Figure 3c.

Figure 4b shows the ultraviolet and near-infrared absorbance curves for the GO with 0.05 mg/mL concentration and the GOS aqueous solution. As the figure shows, GO shows a typical UV-Vis characteristic curve. There are two characteristic peaks in the ultraviolet area (wavelength less than 400 mm): the peak at 230 nm and the shoulder peak at 300 nm, respectively corresponding to the π-π* transition and *n*-π* transition C-C bone [13,26]. The UV-Vis spectra of GOS were similar to those of GO, the same as the results of XRD characterization; due to a load of silicon dioxide, the regular stacking of GO was compromised, thus reducing the intensity of the characteristic peak of the graphite oxide phase for GOS.

The infrared spectrogram of GO in Figure 4c displays the characteristic peak that appeared at 1721 cm^−1^, 1253 cm^−1^, and 1062 cm^−1^ for the GO prepared in the experiment. It proves that C=O, C-O, and C-O-C functional groups were successfully inserted into the GO lamella through oxidization [25,27,28]. A wider and stronger absorption band appeared within 3600–3100 cm^−1^, related to the stretching vibrations of the hydroxyl and water molecules in the GO [29]. The existence of oxygen-containing functional groups made the water molecules enter the interlayer or bond with the oxygen-containing group at the edge in the form of hydrogen bonds, allowing the GO to be more hydrophilic; that is, the GO has better dispersity in water [30]. GOS had more new absorption peaks (Figure 4c). Strong peaks appeared in the vicinity of 1105 cm^−1^ and 794 cm^−1^ related to the asymmetric and symmetric oscillations of Si-O-Si. Furthermore, due to Si-OH stretching, a peak appeared near around 972 cm^−1^, indicating that GO successfully loaded silica dioxide [18,31]. The above results demonstrate the successful synthesis of GO and GOS. In addition, despite the compromise of the characteristic structure of GO after the thin layer of SiO_2_ was loaded, its structure is unspoiled. Thus, it still has potential for applications of two-dimension nanomaterial.

### 3.2. The Dispersed States of GO and GOS in Cement Environment

**Figure 5:**
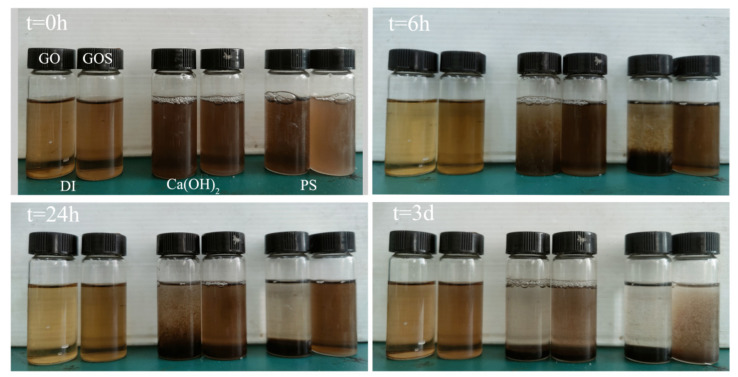
Visual observation of GO and GOS dispersion in different solutions when GO/GOS was added (t = 0 h, 6 h, 24 h, and 3 d).

The dispersed states of GO and GOS in aqueous solution, saturated calcium hydroxide solution, and simulated pore solution underwent macro-observation; the dispersed states are shown in Figure 5. In an aqueous solution, GO and GOS showed better dispersion, and no coagulation occurred after three days; however, GO showed a certain coagulation after being soaked in saturated calcium hydroxide solution containing large amounts of calcium ions and hydroxide ions for 6 h, and a certain degree of stratification was seen at 24 h; after 3 d, it was fully stratified. In terms of GOS, after being soaked in calcium hydroxide saturated solution for 6 h, 24 h, and 3 d, it showed better dispersal than GO. Compared with the dispersed states of GO and GOS in simulated pore solution at different stages, GO stratified before 24 h, and the liquid supernatant became transparent after 3 d. In contrast, distinct coagulation can only be seen after 3 d for GOS.

According to the above test results, GOS has better dispersity in the saturated calcium hydroxide solution and simulated pore solution than GO. Lin et al. [18] attributed the better dispersity to the protection of SiO_2_ on the surface of GO from being crosslinked by divalent calcium ions for the carboxyl on its surface. Furthermore, the SiO_2_ on the surface of GO can undermine the Van der Waals forces of the GO interlamellar layer for its obstructing function, preventing the GOS interlamellar layer from agglomerating due to the inter-attraction of the Van der Waals forces. Comparing the dispersed states of GO and GOS in the saturated calcium hydroxide solution and simulated pore solution revealed that both show better dispersity in the former solution, related to the complicated electrolytes and high alkalinity in the simulated pore solution. According to the study by Zhao et al. [32], the interaction between the complex electrolytes and the PC of the cement pastes enabled the peeling off of the PC attached to the GO and exposed the GO to the environment of Ca^2+^, accelerating the agglomeration of the GO. Through different agglomerations of GO with varying values of pH, Samuel et al. [26] revealed that when such value exceeds 13, the reducing action of GO by sodium and potassium ions and a high alkaline environment will lead to the rapid agglomeration of GO.

### 3.3. The Spreading and Marsh Cone Flow Time with Different Concentrations of GOS

**Figure 6:**
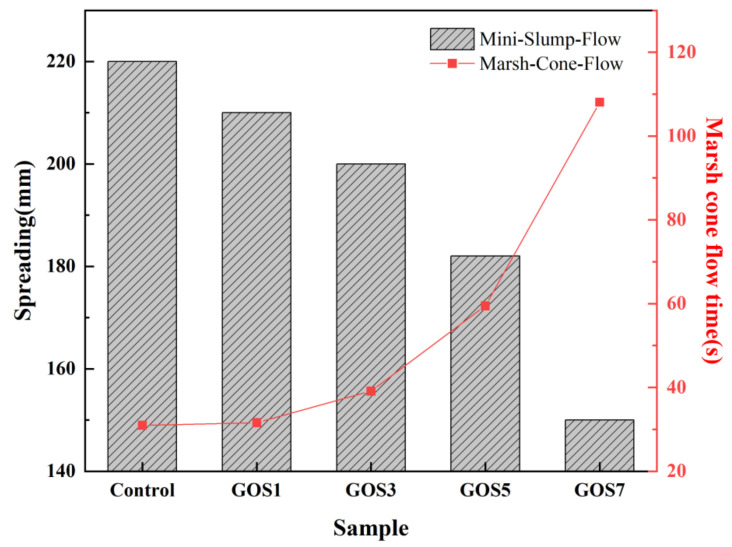
The effects of different GOS concentrations on the spreading of fresh cement paste and Marsh cone flow time.

The addition of nanomaterials to cement-based materials can significantly impact the fluidity of cement pastes. Two simple test methods, spreading and Marsh cone flow time, were adopted to test the fluidity of cement neat pastes in the experiment to explore this phenomenon. Figure 6 shows the spreading and Marsh cone flow time for the reference sample and the samples with different mixing quantities of GOS.

With the increase of GOS, the spreading of cement pastes gradually decreased; however, the Marsh cone flow time gradually increased, which is consistent with the literature [33,34]. When the GOS increased from 0 to 0.07%, the spreading reduced from 220 mm to 150 mm, a decrease of 31.82%. Marsh cone flow time increased by 249.25%, from 30.95 s to 108.09 s; when GOS was increased from 0% to 0.01%, the spreading decreased by 4.54%, while the Marsh cone flow time increased by 2.1%; when it was increased from 0.01% to 0.03%, the former parameter decreased by 4.76% and the latter parameter increased by 23.9%; when it was increased from 0.03% to 0.05%, the former parameter decreased by 9% and the latter bound water parameter increased by 51.97%; when it was increased from 0.05% to 0.07%, the former parameter decreased by 17.58% and the latter parameter raised by 81.7%. In general, with the increase of GOS, the magnitude of spreading gradually grew more extensive, as did the Marsh cone flow time. This tendency may be caused by more water required for the surface moisture due to the increase in the GOS with its high specific surface area. Moreover, the incomplete coverage of SiO_2_ on GO may cause the GOS to be absorbed into the cement particles through the electrostatic interaction. The Van der Waals force between GOS makes the distance between cement particles closer, thus increasing the force of friction between and lowering the fluid performance of the cement pastes [8,35].

### 3.4. The Impacts on the Rheological Properties of Cement Pastes of Different Concentrations of GOS

**Figure 7:**
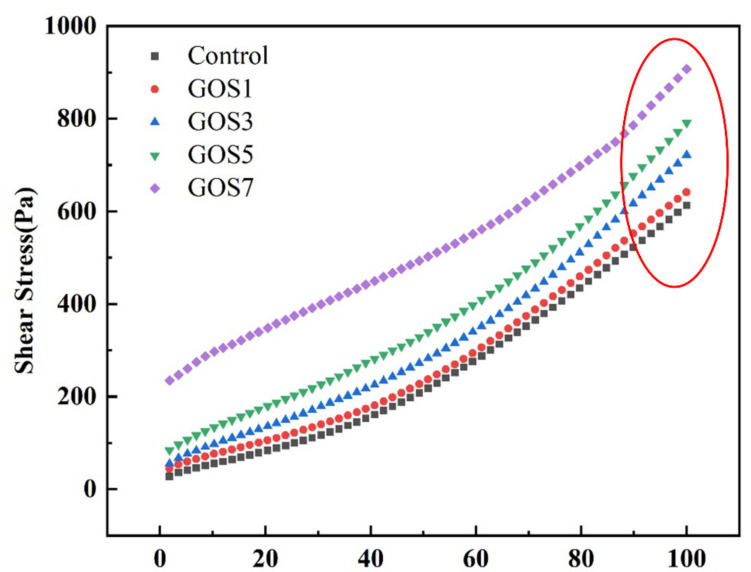
H-B fitting curves of shear rate-shear stress of fresh cement pastes with different GOS concentrations.

Figure 7 shows the shear rate-shear stress curves of fresh cement paste with different GOS concentrations, and the fitting equation is summarized in Table 4. It can be seen from the diagram that the shear stress of fresh cement paste increases with the increase in shear rate. When the shear rate is 100 s^−1^, the shear stress values of the cement pastes with different GOS concentrations are 650, 721, 791, and, 907 Pa. The shear rates of the cement pastes with different GOS concentrations in the reference group are 6.05%, 17.68%, 29.06%, and 48.04%, indicating that the shear stress of the paste increases with the increase in GOS concentration at the same shear rate. This is attributed to the hydrophilicity of GOS, which absorbs part of the free water [36], and when the concentration of GOS increases, it will agglomerate and wrap the free water [11]. These two cases consume the free water in the cement paste, increasing the friction between the cement particles and increasing the shear stress of the cement paste.

**Figure 8:**
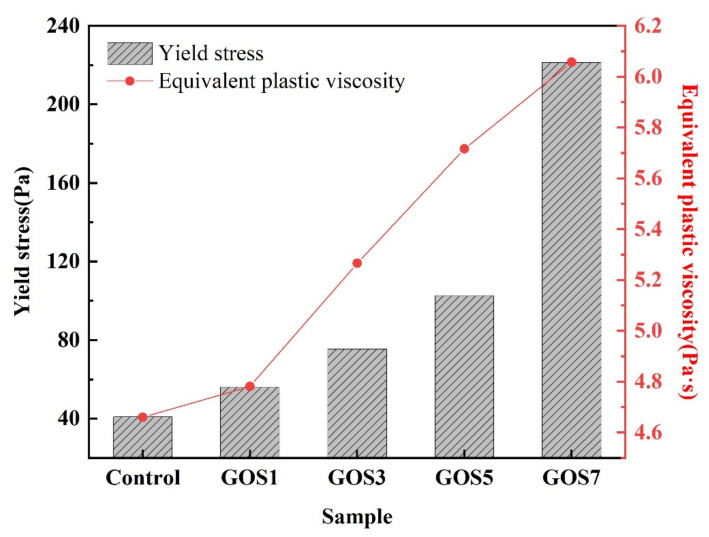
The effects of yield stress and equivalent plastic viscosity of fresh cement paste with different GOS concentrations.

It is well-known that yield stress and equivalent plastic viscosity are the main parameters that affect the rheological properties of cement paste. The paste will deform only when the external force exceeds the yield stress of the cement paste, and the plastic viscosity reflects the speed of the paste deformation. Plastic viscosity is an index to measure the difficulty of material flow. Once the external force on the slurry exceeds the yield stress, it will control the diffusion speed of the paste. The yield stress and plastic viscosity parameters obtained from Equations (1) and (2) are shown in Figure 8. As can be seen from the figure, when the GOS concentration increases from 0 to 0.07%, the yield stress of the corresponding cement paste increases by 37.43%, 84.43%, 151.07%, and 441.54%, indicating that with the rise of GOS concentration, the flow of cement paste becomes more difficult; especially when the GOS concentration is more than 0.05%, the yield stress of cement paste increases more rapidly. In cement paste, segregation is mainly caused by uneven flow and gravity settlement between the components of the fresh mixture, and viscosity is the main parameter for evaluating the segregation of cement paste [37]. According to Figure 8, with the increase of GOS concentration, the equivalent plastic viscosity of cement paste increases by 2.62%, 13.00%, 22.65%, and 29.97%. On the whole, the addition of GOS will increase the plastic viscosity of cement paste, slow down the flow speed of cement paste, and reduce the probability of cement paste segregation.

**Figure 9:**
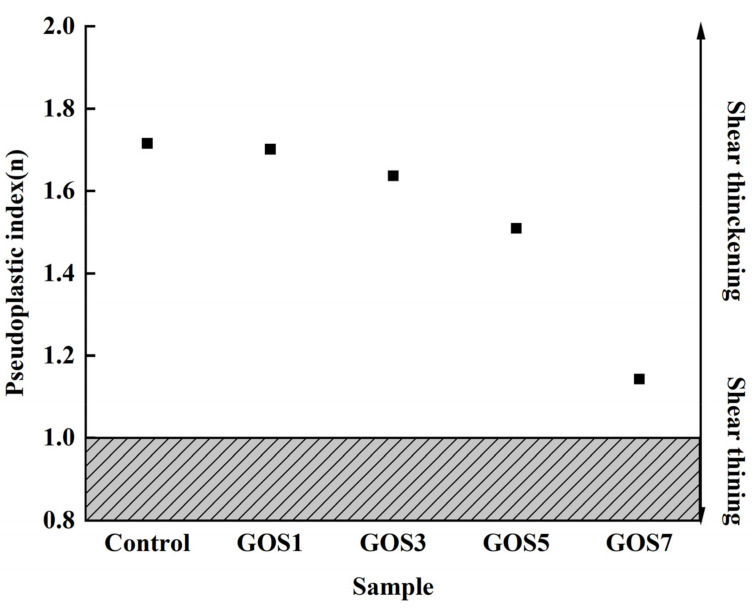
The changes in the pseudoplasticity index of fresh cement pastes with different GOS concentrations.

Figure 9 shows the effects of different GOS concentrations on the pseudoplasticity index (*n*) of fresh cement paste. As shown in Figure 9, the rheological index *n* of fresh cement paste fitted by H-B is greater than 1, indicating that the fresh cement paste mixed with superplasticizer shows shear thickening under shear action, but the addition of GOS will not change this property. With the increase of GOS concentration, the *n* value of fresh cement decreased gradually, and the decreasing rates were 0.82%, 4.56%, 12.00%, and 33.35%. The results show that the *n* of fresh cement decreases and increases with the increase of GOS concentration. This is related to the interaction between GOS, PC, and cement particles. Previous studies have shown that PC will produce chemical and physical adsorption to cement particles, and the long side chain of PC will cause steric hindrance between cement particles, thus increasing the distance between cement particles, while the increase of distance will reduce the electrostatic repulsion between cement particles. There is also a certain interaction between GOS and PC that will reduce the adsorption of PC to cement particles, reduce the distance between cement particles, and increase the electrostatic repulsion between cement particles. With increasing GOS concentration, the steric hindrance between cement particles decreases, and the repulsive force between cement particles increases gradually. According to the cluster theory, when the hydrodynamic force is greater than the repulsive force between the particles, the cement particles will form a cluster structure, which leads to the shear thickening of the cement paste. Therefore, the higher the concentration of GOS, the greater the repulsive force between cement particles, and the more difficult it is for cement paste to form shear thickening, so the pseudoplasticity index of cement paste will decrease with the increase of GOS concentration [35,38,39].

### 3.5. The Impacts on the Mechanical Properties of Cement-Based Materials with Different Concentrations of GOS

**Figure 10:**
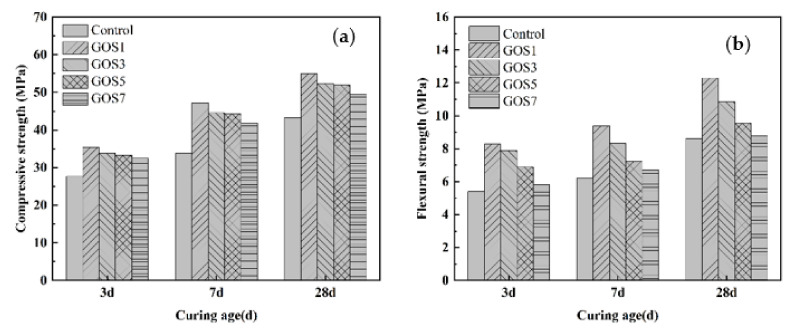
Strengths of cement-based materials with different GOS concentrations: (**a**) Compressive Strength. (**b**) Flexural strength.

Figure 10a,b displays the compressive and flexural strengths of cement samples with different concentrations of GOS at the maintenance periods of 3 d, 7 d, and 28 d. According to Figure 10a, at different maintenance periods, the compressive strength of cement samples varies with the addition of GOS. In contrast to the reference group, the compressive strength for the cement sample at 3 d increased by 27.73%, 22.38%, 20.07%, and 17.35%; at 7 d of curing, the number increased by 39.26%, 31.67%, 31.02%, and 23.40%; and at 28 d of curing, it increased by 27.17%, 20.74%, 19.95%, and 14.31%. According to Figure 10b, in comparison with the reference group, the flexural strength of cement samples with different concentrations of GOS increased by 53.70%, 46.11%, 27.59%, and 7.41% at 3 d of curing; the number increased by 50.72%, 33.71%, 15.89%, and 7.87% at 7 d of curing, and it increased by 42.86%, 26.13%, 11.15%, and 1.86% at 28 d of curing. All of the results indicate that the strengthening effect of GOS on the mechanical properties of cement-based composites will not show a linear tendency along with increasing concentrations of GOS; instead, there is an optimal concentration, and beyond the optimal range, the strength has a downward trend. The template action and accelerated hydration of GOS in the cement-based materials allow more and tighter hydration products in such materials, thus improving their compressive strength [40,41]. However, with the additional increase of GOS, aggregation occurs; as a result, cracks will be seen due to the failure of the original effect of nanomaterials, and the strength will decrease because of the concentrated stress [42]. Moreover, it can be determined that the increasing magnitudes at 7 d and 3 d are greater than that of 28 d by comparing the compressive strength of GOS cement-based materials on 3 d, 7 d, and 28 d. The evenly distributed GO in the early stage will promote the generation of hydration products because more nucleation sites and the generated C-S-H gel from the pozzolanic reaction of SiO_2_ with Ca (OH)_2_ will accelerate the hydration [18,43].

### 3.6. The Impacts on the Microstructure of GOS to Cement-Based Materials

#### 3.6.1. The X-ray Diffractometer Analysis

**Figure 11:**
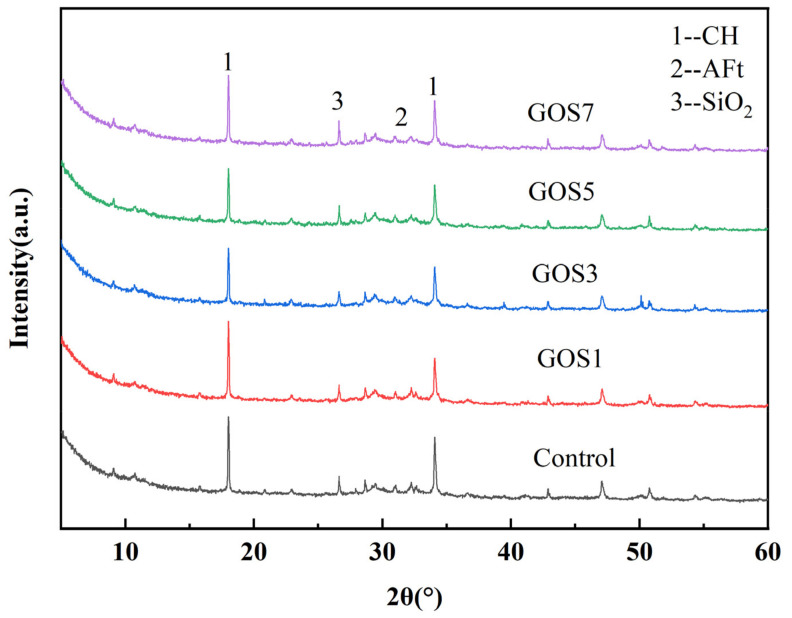
XRD patterns of cement-based materials with different GOS concentrations.

The impacts on the hydration products of cement-based materials with different concentrations of GOS in 28 d were analyzed through X-ray diffractometer. As shown in Figure 11, compared with the reference group, the XRD spectra of the cement-based materials with the addition of GOS show no new crystal orientation and no alteration of the diffraction peak position, indicating that GOS has no impact on the constitution of the hydration products of cement. However, it is worth noting that when the peak intensity at 2θ is 18° of the CH (Ca (OH)_2_) main peak, the addition of GOS will affect the formation of CH in the cement-based materials. With the additional increase of GOS, the peak intensity for CH will first decrease and then increase. Compared with the control group, the intensity of the CH peak decreased gradually with the increase of GOS concentration, but this is because the SiO_2_ attached to the GOS layer will react with CH in the middle and later stages of hydration. Therefore, when the concentration of GOS increases, the content of CH decreases gradually [44]. However, when the concentration of GOS increases to 0.07%, the GOS in the system agglomerates, which reduces the contact between SiO_2_ and CH, thus reducing the consumption of CH by SiO_2_, so the CH peak strength of the GOS7 cement matrix is increased.

#### 3.6.2. TG-DTG Analysis

**Figure 12:**
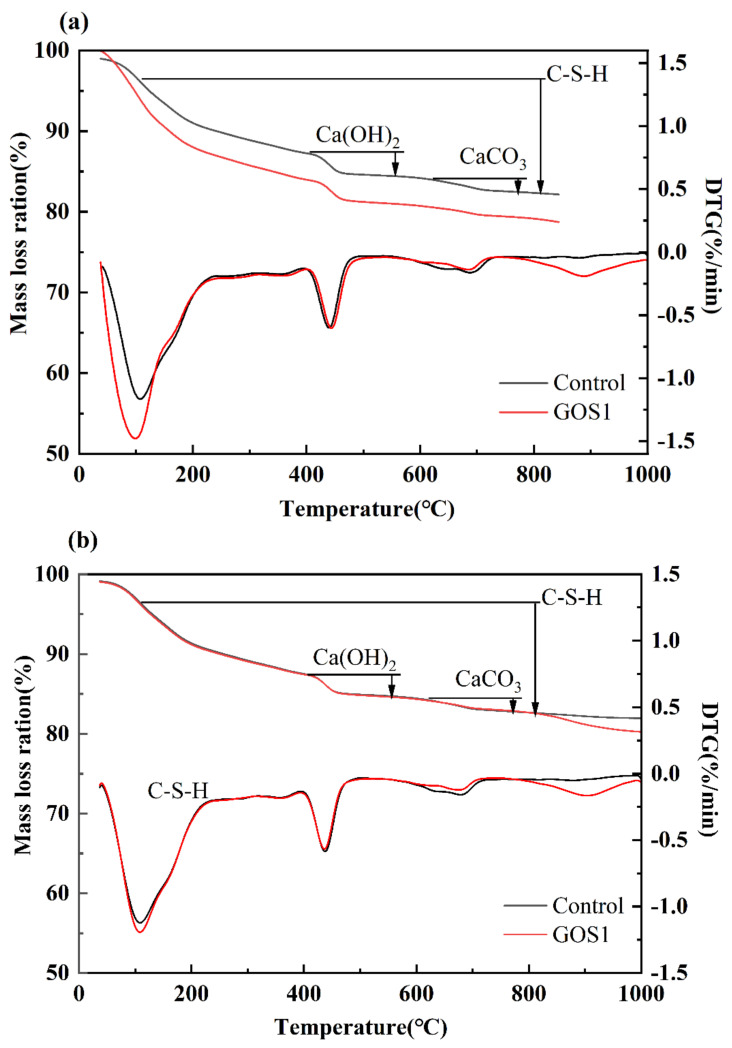

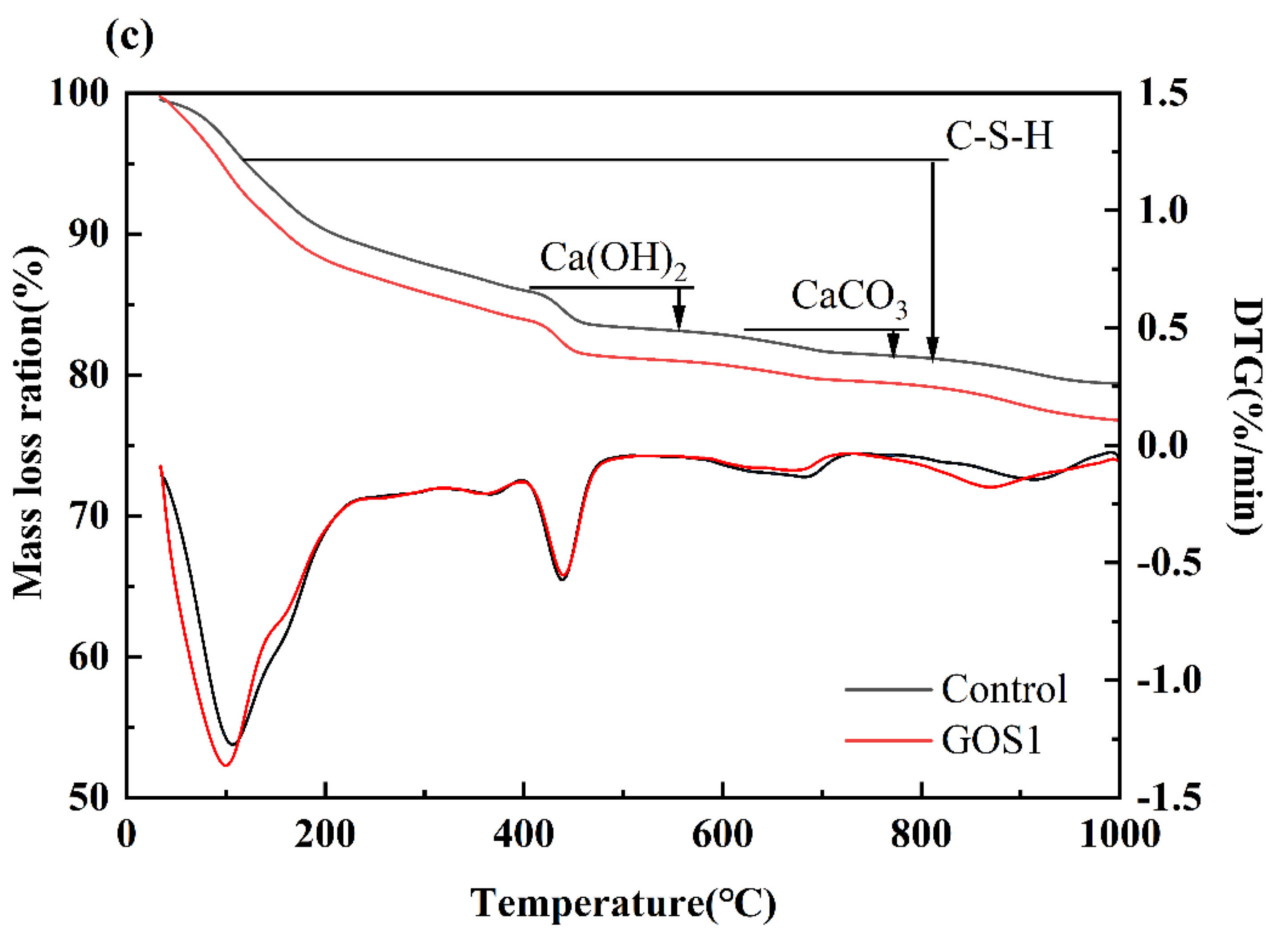
The TG−DTA curves of the control group and the GOS1 experimental group at different hydration ages: (**a**) 3 d. (**b**) 7 d. (**c**) 28 d.

The TG-DTG curves are shown in Figure 12. The curves show that there are mainly three distinct decomposing endothermic peaks during the entire temperature rising course. The peak value near 105 °C is mainly the endothermic evaporation of unbound water. At around 450 °C is the endothermic peak for the decomposition of CH_,_ and the peak at 600–700 °C was generated by the decomposition of CaCO_3_ in the carbonized sample. Comparing the reference group with the endothermic peak for GOS1 at different periods, it turns out that the endothermic evaporation peak of GOS1 chemical bound water is better than the reference group, indicating that the addition of GOS has promoted the generation of ettringite and C-S-H gel. In terms of the endothermic peak of CH and CaCO_3_, the endothermic peak of the reference group is slightly higher than that of the GOS1 group in 7 d and 28 d, which is consistent with the results of XRD characterization. It indicates that the SiO_2_ covering the GO surface generates the pozzolanic reaction with CH, consuming a certain amount of CH.

The composition of hardened cement-based materials consists of hydration products, porous water, and unhydrated cement. Among these, the hydration products comprise chemically bound water, which contains non-evaporative water and the weak bond water in the ettringite and the C-S-H gel. It is generally believed that the mass loss at 105–800 °C refers to the loss of chemically bound water, but the one at 400–500 °C and 600–700 °C refers to the endothermic decomposition of CH and CaCO_3_ [45,46]. The mass loss data of specific temperatures have been intercepted to calculate the chemically bound water and CH content for the GOS1 group in different periods according to Equations (3) and (4). The results can be seen in Table 5.

According to the calculation results, the BW content of GOS1 cement-based material was higher than that of the reference group at 3 d, 7 d, and 28 d. This indicates that GOS synthesized by the sol-gel method can effectively accelerate the early hydration reaction and promote the production of more C-S-H gels. The calculation results of CH content are shown in Table 5. At 3 d, the CH content of GOS1 was higher than that of the reference group; at 7 d and 28 d, the CH content of GOS1 was lower than that of the reference group. GOS contains SiO_2_, which has pozzolanic activity and can react in the middle and late stages, consuming CH in the cement matrix. Therefore, in the early stage, GOS can accelerate the hydration reaction to produce more CH. Over time, CH is absorbed by SiO_2_ and thus reduces the CH content in the cement matrix. This is consistent with the previous characterization of CH by XRD.

#### 3.6.3. SEM Analysis

**Figure 13:**
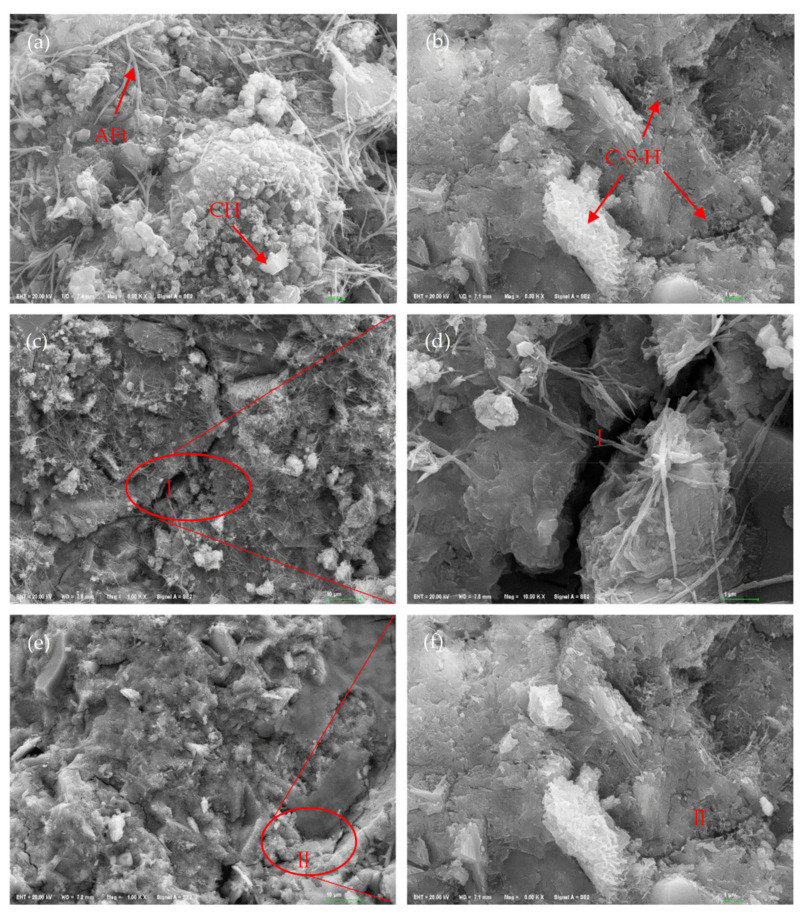
SEM pictures of cement-based materials cured for 28 d: (**a**,**c**,**d**) Control. (**b**,**e**,**f**) GOS1.

The cement-based materials of the 28 d reference sample and GOS1 sample were characterized by SEM. The results are shown in Figure 13. The loose accumulation of CH tetragonal crystals and AFt needle-like crystals can be observed in the reference samples (Figure 13a). The loose structure can easily lead to crack initiation and propagation. As shown in Figure 13c,d, the cracks in zone I are not effectively suppressed, resulting in propagation, which reduces the mechanical properties of cement-based materials. In the GOS1 sample (Figure 13b), the structure of C-S-H and ettringite increased clearly and became denser. In addition, the secondary hydration reaction between SiO_2_ and CH further increased the content of C-S-H, which made the C-S-H show good fusion. This is consistent with the characterization results of XRD and TG-DTG. In addition, more well-integrated hydration products can become a barrier crack propagation and thereby effectively improve the mechanical properties of cement-based materials [47]. As shown in area II of Figure 13e,f, the addition of GOS to cement-based materials promotes the formation of C-S-H, significantly improves the microstructure of cement-based materials, and effectively restrains the crack propagation by nano-filling and the bridging effect.

### 3.7. The Comparison of Macro Performance for Cement-Based GOS and GO Materials

To determine the improvement of GOS to GO, the flow property, rheological parameters, and mechanical properties of GO and GOS with the same addition (0.01%) were compared.

**Figure 14:**
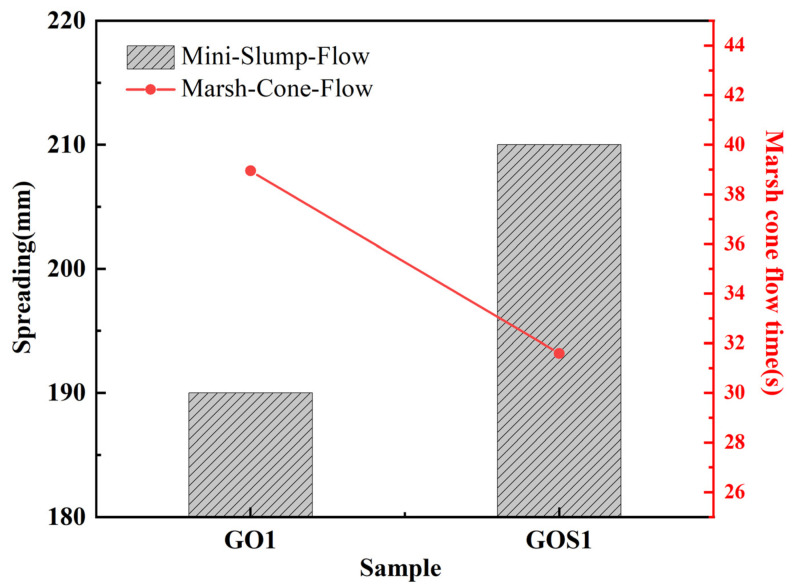
Spreading and marsh cone flow times of GO and GOS (0.01%) cement-based materials.

**Figure 15:**
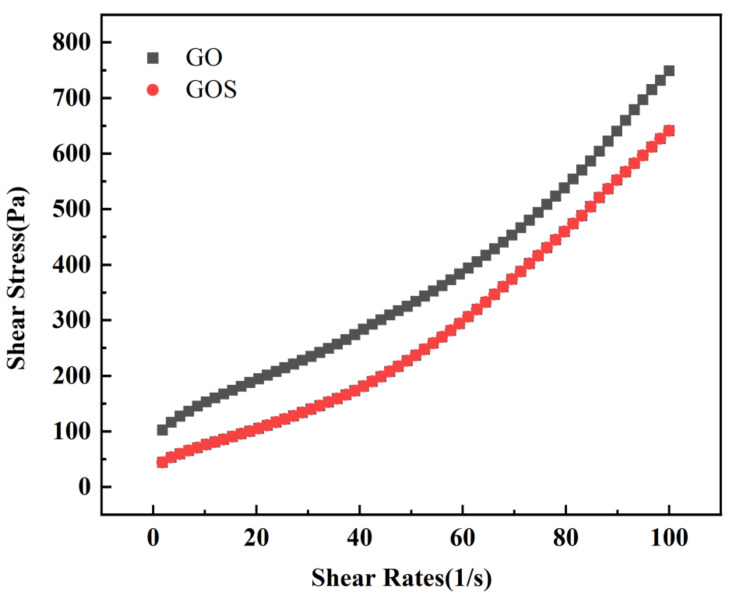
Shear rate-shear stress H-B fitting curves of GO and GOS (0.01%) cement-based materials.

**Figure 16:**
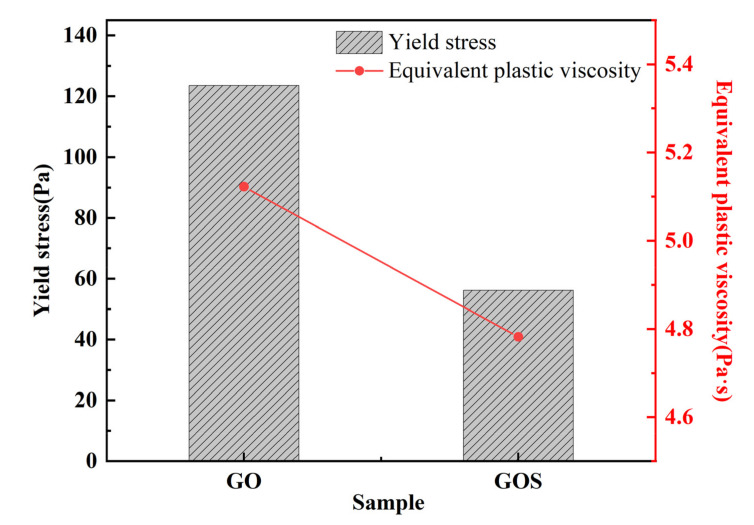
Yield stress and equivalent plastic viscosity of GO and GOS (0.01%) cement-based materials.

Figure 14 illustrates the comparison of spreading and Marsh cone flow times between GOS1 and GO1. According to the test results, compared with samples with the addition of GO, the spreading of cement pastes containing GOS increased by 10.53%, and the Marsh cone flow time decreased by 18.9%. Figure 15 shows the shear rate-shear stress curves of GO1 and GOS1, with GO1 above GOS1. The figure indicates that the shear stress to drive the cement-based materials of GO1 is higher with the same shear rate. Figure 16 and Table 6 present the rheological parameters of the GO1 and GOS1 cement pastes. Compared with GO, the shear yield stress of GOS decreased by 54.54%. The equivalent plastic viscosity decreased by 6.65%, indicating that the SiO_2_ covering the GO improved the fluidity of the GO cement pastes, thus, lowering the rheological parameters of the cement pastes, which was consistent with the study results of Lin et al. [18]. The hydrophilic SiO_2_ covering the GO effectively prevented the complexation of GO and Ca^2+^. Meanwhile, the obstructing function of SiO_2_ reduced the physical absorption between GOS and cement particles because of the electrostatic interaction. As a result, the force of friction among cement particles reduced, the flow properties of the cement pastes improved, and its rheological parameters decreased [8,9]. According to Table 6, the pseudoplasticity index of GOS cement paste is higher than that of GO, which indicates that the flocculation structure formed in GO cement paste is relatively loose, which is easy to be dispersed under the action of shear force, which leads to the decrease of pseudoplasticity index, while the flocculation structure formed in GOS cement paste is closely related and not easy to break, so the pseudoplasticity index is relatively high [48].

**Figure 17:**
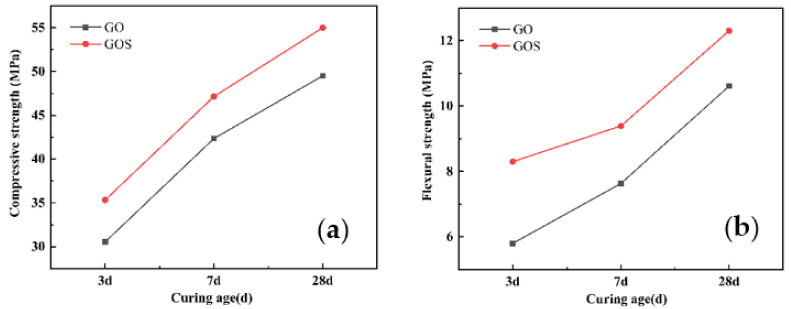
The strengths of the GO and GOS cement-based materials (0.01%): (**a**) compressive strength, (**b**) flexural strength.

According to Figure 17, compared with GO1 specimens, the compressive strength of GOS1 cement-based materials increased by 15.68%, 11.31%, and 11.11% after 3 d, 7 d, and 28 d, respectively; flexural strength increased by 43.1%, 23.07%, and 15.93%, respectively. This shows that the GOS can improve the flexural properties of GO cement-based materials. According to the previous discussion, the formation of GOS on the surface of GO coated with SiO_2_ can effectively improve the dispersion of GO in cement-based materials, and the excellent dispersion can give full play to the nano-filling effect of GOS. Therefore, in the middle and later stages of hydration, the GOS that fills in the pores will consume CH to produce a secondary hydration reaction, resulting in more C-S-H gel. On the other hand, the C-S-H gel grown on the GOS layer is tightly connected, which can effectively prevent the propagation of microcracks and effectively improve the bending strength of cement-based materials [33].

### 3.8. The Comprehensive Evaluation of the Impacts of GO and GOS on Cement-Based Materials through the Fuzzy Matrix

**Figure 18:**
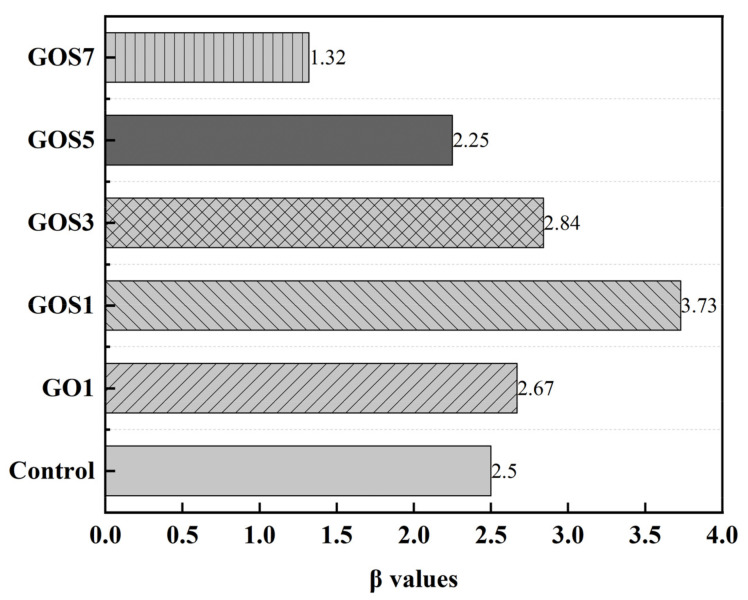
Bar chart of the comprehensive evaluation scores of the fuzzy matrix.

The fuzzy mathematical matrix was adopted to compare further and analyze the impact of GO and GOS to cement and its comprehensive performance [49]; the main steps are shown below:

1. The matrix evaluation factors should be determined, F = {F_1_, F_2_, F_3_, F_4_, F_5_} = {F_1_(spreading), F_2_ (yield stress), F_3_ (equivalent plastic viscosity), F_4_ (compressive strength of 28 d), F_5_ (flexural strength of 28 d)}.
2. The evaluation matrix should be determined, U = {u_1_, u_2_, u_3_, u_4_} = {u_1_ (very good), u_2_ (good), u_3_ (general), u_4_ (poor)}, and the corresponding grade should be given respectively: α_1_ = 4, α_2_ = 3, α_3_ = 2, α_4_ = 1. The maximum and minimum values can be determined by the above performance tests. The set of evaluation factors and the standard evaluation value of the evaluation collection of fuzzy evaluation can be determined in an arithmetic sequence, as shown in Table 7.
3. The comprehensive evaluation matrix R_5×4_ was determined based on the grade of membership of comments assemble to the performance indexes of cement-based materials with different contents GO and GOS, the corresponding compressive evaluation matrix can be obtained as
  $$\begin{array}{cc} R_{C}=\left[\begin{array}{cccc} 1 & 0 & 0 & 0\\ 1 & 0 & 0 & 0\\ 1 & 0 & 0 & 0\\ 0 & 0 & 0 & 1\\ 0 & 0 & 0 & 1 \end{array}\right] & R_{GO1}=\left[\begin{array}{cccc} 0 & 0.778 & 0.223 & 0\\ 0 & 0 & 0.903 & 0.097\\ 0 & 0.684 & 0.316 & 0\\ 0 & 0.012 & 0.988 & 0\\ 0 & 0.920 & 0.080 & 0 \end{array}\right]\\ R_{GOS1}=\left[\begin{array}{cccc} 0.333 & 0.667 & 0 & 0\\ 0.386 & 0.614 & 0 & 0\\ 0.583 & 0.417 & 0 & 0\\ 1 & 0 & 0 & 0\\ 1 & 0 & 0 & 0 \end{array}\right] & R_{GOS3}=\left[\begin{array}{cccc} 0 & 0.667 & 0.333 & 0\\ 0 & 0.796 & 0.203 & 0\\ 0 & 0.417 & 0.583 & 0\\ 0.058 & 0.942 & 0 & 0\\ 0.08 & 0.92 & 0 & 0 \end{array}\right]\\ R_{GOS5}=\left[\begin{array}{cccc} 0 & 0 & 0.889 & 0.111\\ 0 & 0.221 & 0.779 & 0\\ 0 & 0 & 0.603 & 0.397\\ 0 & 0.934 & 0.066 & 0\\ 0 & 0.256 & 0.744 & 0 \end{array}\right] & R_{GOS7}=\left[\begin{array}{cccc} 0 & 0 & 0 & 1\\ 0 & 0 & 0 & 1\\ 0 & 0 & 0 & 1\\ 0 & 0 & 0.995 & 0.005\\ 0 & 0 & 0.286 & 0.714 \end{array}\right] \end{array}$$
4. The weight is highly crucial in fuzzy evaluation. It refers to the relative importance of the performance index in the comprehensive evaluation. In this paper, cement-based materials’ rheological properties, compressive strength, and flexural strength are the important performance indexes. Therefore, the weighing matrix will be w = {0.1, 0.2, 0.2, 0.25, 0.25}.
5. The evaluation results matrix B = w × R can be obtained from the comprehensive evaluation matrix and the weighing matrix; then, the comprehensive evaluation results can be obtained through the equation; Figure 18 shows the scores.

The properties of GO and GOS cement-based materials were evaluated comprehensively by the fuzzy matrix method. Compared with GO1 cement-based materials, GOS1 cement-based materials have larger spreading, lower yield stress, and equivalent plastic viscosity; in addition, GOS1 cement-based materials also have a certain improvement in mechanical properties, especially the greatest increase in flexural strength. The comprehensive properties of GOS cement-based materials did not increase with the increase of GOS content. With the increase of GOS content, the spreading degree of cement-based materials decreased, the yield stress and equivalent plastic viscosity increased, and the improvement of mechanical properties also decreased. The final scores of the comprehensive evaluation are GOS1 > GOS3 > GO1 > Control > GOS5 > GOS7, indicating that the cement-based material with GOS1 added has better comprehensive performance.

## 4. Conclusions and Prospect

To further improve the dispersity of GO in cement-based materials, the sol-gel method was adopted to synthesize a novel two-dimension GO-SiO_2_ (GOS) hybrid material in this paper. First, the prepared GO and GOS were characterized microscopically, and the dispersibility of GO and GOS in different solutions were compared and analyzed. Second, the effects of GOS content on the mechanical properties, rheological properties, and microstructures of cement-based materials were studied. Third, the macroscopic properties of GO and GOS cement-based materials with 0.01% content were compared and analyzed. Lastly, the fuzzy matrix method was adopted to evaluate and analyze the comprehensive performance, and the following conclusions have been reached:

1. The newly prepared GOS nano hybrid materials preserve the two-dimension material feature of GO. The evenly attached nano SiO_2_ on the surface of GO was conducive to the dispersity stability in the calcium hydroxide saturated solution and the simulated pore solution.
2. The spreading of the cement-based materials decreased gradually with the increase in GOS concentration, while the Marsh cone flow time shows an increasing tendency. When the GOS concentration was 0.01%, the above parameters corresponded to 210 mm and 31.59 s.
3. The GOS can distinctly enhance the mechanical properties of cement-based materials. When the GOS content was 0.01%, compared with the reference sample, the compressive and flexural strength increased by 27.17% and 42.86% on 28 d.
4. With the same addition, the spreading degree of GOS1 cement-based materials is higher than that of GO1. The shear yield stress and equivalent plastic viscosity are lower than those for the GO1 cement-based materials. In terms of compressive and flexural strength at 28 d, compared with the GO1 cement-based materials, they were 11.11% and 15.93% higher.
5. According to the evaluation results of fuzzy matrix comprehensive performance, the addition of 0.01–0.03% of GOS can improve the comprehensive performance of cement-based materials, with the highest score when the added GOS was 0.01%, indicating the best comprehensive performance at 3.73.

The synthesis process of the hybrid material is simple, the time required is short, and the process has the potential to realize large-scale preparation. While ensuring the good fluidity of cement paste, it improves the mechanical properties of the cement-based materials, avoids the dispersion and fluidity problems encountered in the application of GO in cement-based materials, and takes a big step forward for the application of GO in practical engineering.

## Figures and Tables

**Table 1:** Chemical composition of cement power.

CaO	SiO_2_	Al_2_O_3_	Na_2_O	K_2_O	Fe_2_O_3_	SO_3_	MgO	P_2_O_5_	L.O.I
58.18	21.25	5.67	0.2	0.62	3.16	3.66	2.41	0.09	3.95

**Table 2:** Mix design for cement paste.

Sample	w/c	Nanomaterial (by Weight of Cement)	PC (by Weight of Cement)
Control	0.4	/	0.2%
GO1	0.4	0.01% GO	0.2%
GOS1	0.4	0.01% GOS	0.2%
GOS3	0.4	0.03% GOS	0.2%
GOS5	0.4	0.05% GOS	0.2%
GOS7	0.4	0.07% GOS	0.2%

**Table 3:** The chemical composition of the pore solution.

Component	Ca(OH)_2_	CaSO_4_	NaOH	KOH
Concentration (g/L)	Saturated	27.6	8.2	22.4

**Table 4:** The fitting curve equation of the cement paste obtained by H-B fitting.

Sample	The Fitting Equation	*n*	R^2^
Control	y = 40.87 + 0.21×^1.72^	1.72	0.99957
GOS1	y = 56.16 + 0.23×^1.70^	1.70	0.99738
GOS3	y = 75.38 + 0.34×^1.63^	1.63	0.99554
GOS5	y = 102.61 + 0.64×^1.51^	1.51	0.99274
GOS7	y = 221.32 + 3.28×^1.14^	1.14	0.96706

**Table 5:** Chemical binding water and CH content of reference sample and GOS sample at different ages.

Sample	3 d		7 d		28 d	
	BW	CH	BW	CH	BW	CH
Control	13.39%	13.11%	14.35%	14.88%	15.31%	15.74%
GOS1	14.69%	14.92%	15.15%	13.53%	17.12%	14.23%

**Table 6:** H-B fitting curve equations of cement-based materials with the same GO and GOS contents.

Sample	The Fitting Equation	*n*	R^2^
GO	y = 123.53 + 0.51^1.53^	1.53	0.98758
GOS1	y = 56.16 + 0.23^1.70^	1.70	0.99738

**Table 7:** Fuzzy evaluation standard values based on testing results.

	v_1_ (Very Good)	v_2_ (Good)	v_3_ (Fair)	v_4_ (Poor)
u_1_ (spreading), mm	220.00	205.00	190.00	150.00
u_2_ (yield stress), Pa	40.87	61.56	88.99	221.32
u_3_ (viscosity), Pa·S	4.66	4.80	5.49	6.06
u_4_ (compressive strength at 28 d), MPa	55.00	52.05	49.47	43.25
u_5_ (flexural strength at 28 d), MPa	12.30	10.74	9.17	8.61

## Data Availability

The data presented in this study are available on request from the corresponding author.
